# Feasibility of ^125^I brachytherapy combined with arterial infusion chemotherapy in patients with advanced pancreatic cancer

**DOI:** 10.1097/MD.0000000000035033

**Published:** 2023-11-03

**Authors:** Shujing Huang, Yanqing Cao, Rui Wang, Huimin Liu, Ting Wang, Shu Yang

**Affiliations:** a Department of Oncology, The First Affiliated Hospital of Guangdong Pharmaceutical University, Guangzhou, Guangdong, China.

**Keywords:** ^125^I brachytherapy, advanced pancreatic cancer, arterial infusion chemotherapy

## Abstract

To evaluation the feasibility of Iodine-125 (¹²^5^I) brachytherapy combined with arterial infusion chemotherapy in patients with advanced pancreatic cancer. A total of 72 cases with Stage III and IV were retrospectively reviewed. 23 cases receiving ^125^I brachytherapy were classified as Group A. 27 cases receiving arterial infusion chemotherapy (gemcitabine + cisplatin, GP) were classified as Group B and 22 cases receiving ^125^I brachytherapy combined with arterial infusion chemotherapy (GP) were classified as Group C. The evaluated indications were local control rate, survival rate, carbohydrate antigen 19-9, pain relief, and Karnofsky physical scores. Analysis of Variancep, Pearson chi-square test and Kaplan–Meier curves were used for analysis. The local control rate of group A and group C was significantly higher than group B (*P* < .001). Pearson chi-square test showed statistical difference of the 3 groups (χ^2^ = 12.969, *P* = .044). The median survival of group A,B and C was 9 months, 6 months and 13 months, respectively. The survival time of group C was significantly higher than group B (χ^2^ = 5.403, *P* = .020). The Log rank test showed statistical difference in the survival curve of the 3 groups (χ^2^ = 6.501, *P* = .039). The difference of carbohydrate antigen 19-9 decline percentage between group B and C group was statistically significant (χ^2^ = 5.959, *P* = .015). Patients in group A and group C relieved form pain after treatment with statistically significant (*P* < .001). Pain relief was much more effective in patients who received ^125^I brachytherapy. Karnofsky physical scores after treatment were statistically higher than those before treatment in each group (*P* < .001). ^125^I brachytherapy maybe one of the effective, safe and feasible alternative treatment of advanced pancreatic cancer. ¹²^5^I brachytherapy combined with arterial infusion chemotherapy was effective in the treatment of advanced pancreatic cancer.

## 1. Introduction

Pancreatic cancer is one of the aggressive malignancy with the worst prognosis, of which the morbidity and mortality is increasing significantly in recent years.^[1–3]^ The treatment is mainly surgery that combined with radiotherapy and chemotherapy. Since its early symptoms are not obvious, many patients lose the opportunity of surgery. Even if the cancer is discovered early, only 20% of patients can undergo surgical excision, whereas the other 80% cannot do.^[4,5]^ It is not sensitive to the chemotherapy and external radiotherapy.^[6]^ Overall survival (OS) was not prolonged by chemoradiotherapy in advanced pancreatic cancer, the 5-year survival rate was still 5%.^[7]^

In recent years, there is no standard therapy for advanced pancreatic cancer in National Comprehensive Cancer Network Guideline. The clinical application of Iodine-125 (^125^I) is becoming more and more wider. ^125^I release low dose of X-rays and γ-rays continually, which ionize water molecules, produce free radicals, break DNA double strand by acting on DNA directly and promote apoptosis of tumor cells. Its half-life is 59.6 days. Its radiation total dose can reach to 110 to 160 Gy, strongly and precisely, with a half-life of 59.6 days and a radiation diameter of 1.7 cm. While, the radiation dose outside the radioactive sources can decay quickly, with less damage on the surrounding normal tissues and better protection of the normal tissues.^[8–10]^ Intraoperative radiotherapy is another type of brachytherapy. However, it is only suitable for patients willing to undergo surgery. Arterial infusion chemotherapy is another form of minimally invasive treatment for tumors. It is delivered directly by selective catheterization into the target artery feeding the tumor.^[11–14]^ Gemcitabine with cisplatin (GP) is one of the chemotherapy of first-line therapy of advanced pancreatic cancer. The rationale for arterial infusion chemotherapy can achieve increased local drug concentrations in the tumor to the levels which can not be achievable by intravenous systemic treatment, meanwhile reducing side effects.^[13,14]^

This study investigated the efficacy of ^125^I brachytherapy combined with arterial infusion chemotherapy in the advanced pancreatic cancer, and analyzed the complications and feasibility.

## 2. Methods

### 2.1. Patients

Between January 2012 and January 2020, a total of 72 patients who were diagnosed with advanced pancreatic cancer were reviewed in the study. Advanced pancreatic cancer was defined as having local and/or distant metastases that cannot be surgically resected. Before treatment initiation, all subjects were fully aware of the potential risks and provided written informed consent. Written informed consent of the study was waived because it was a retrospective study, and patient data were kept strictly confidential.

The inclusion criteria: pancreatic cancer confirmed by pathologic diagnosis; the Stage III (tumor invading the celiac axis or the superior mesenteric artery with or without spreading to lymph nodes) or Stage IV (metastasis) (based on the American Joint Committee on Cancer TNM Staging of Pancreatic Cancer, 8th ed., 2017) that was ineligible for surgical resection; Karnofsky physical scores (KPS) ≥ 60; no systemic infection; and white blood cell ≥3 × 10^9^/L, platelets ≥100 × 10^9^/L, and hemoglobin ≥90 g/L, no severe coagulation disorders in peripheral blood. The rejection criteria were as follows: patients had dysfunction of vital organs such as heart, lung, liver, kidney or cachexia; patients with severe jaundice should treat jaundice first; combined with other cancers or compound carcinoma; other anti-cancer treatments within 2 months; patients with upper gastrointestinal bleeding recently or other potential serious diseases that interfered with this study; patients lack of detailed clinical information or loss to follow-up.

Within 2 weeks prior to treatment, a pancreatic computed tomography (CT) enhancement scan was performed on all patients. Total 23 patients received the therapy of CT-guided ^125^I brachytherapy (group A). 27 patients were treated with arterial infusion chemotherapy (group B). 22 patients received the combined therapy of CT-guided ^125^I brachytherapy combined with arterial infusion chemotherapy (group C). All patients of group C were treated with arterial infusion chemotherapy 3 or 4 days after implantation of ^125^I, with the same chemotherapy as that of group B. The baseline-characteristics of the patients are shown in Table 1.

**Table 1:** General information of the patients.

Data	^125^I brachytherapy		Arterial infusion		^125^I brachytherapy + arterial infusion		X^2^	*P* value
	Cases	%	Cases	%	Cases	%		
No. of patients	23	100	27	100	22	100		
Median age (range)	63 (40–82)		57.5 (29–74)		56.5 (43–78)		1.294	.281
Sex							1.804	.406
Male	13	56.5	15	55.6	16	72.7		
Female	10	43.5	12	44.4	6	22.2		
Position of lesion							0.064	.968
Head	10	43.5	12	44.4	9	40.9		
Body and tail	13	56.5	15	55.6	13	59.1		
Diameter of lesion (largest)(cm)	2.9–7.5		2.0–7.4		2.1–7.8			
Clinical stage							0.518	.772
Stage IV	17	73.9	20	74.1	18	81.8		
Stage III	6	26.1	7	25.9	4	18.2		
Clinical manifestations before treatment							1.952	.745
Jaundice	7	30.4	3	11.1	4	18.2		
Pain	19	82.6	20	74.1	20	90.9		
Elevated CA19-9	20	87.0	22	81.5	20	90.9		

125 I = iodine-125, CA19-9 = carbohydrate antigen 19-9.

### 2.2. Instruments

We used a high-speed advantage genesis CT scanner (Philips) that set at 120 kVs, 265 mA, 5-mm layer thickness.^125^I seeds (6711/BT-^125^I) were manufactured by Beijing Atom and High Technology Industries Inc (Beijing, China). The ^125^I seed was a cylindrical titanium alloy package, with the length of 4.5 mm, the diameter of 0.8 mm, the radiation diameter of 1.7 cm, the activity of 0.8 mCi and the half-life of 59.6 days. The ^125^I seeds were implanted by 18-G implantation needles and a turntable implantation gun. The treatment planning system (TPS), which was designed by the Beijing University of Aeronautics and Astronautics, was used in preoperative ^125^I seed implantation planning and postoperative dose verification. Angio-catheters were manufactured by Terumo Medical Products Company Limited (Hangzhou, China).

### 2.3. ^125^I seed implantation

Before the implantation of seed, the professional physicist sends CT image of the patient to the TPS to reconstruct the 3-dimensional digital image and draw target delineation. According to 3 mutually perpendicular diameters of tumor target volume, the total radiation dose, particle radioactivity, spatial distribution of the particles and tumor matching peripheral dose (MPD) were calculated to determine the location and direction of needle implantation, and determine the number of implanted particles. Patients ate liquid diet 2 days before the operation, and fasted for 24 hours prior to the operation, and oral laxatives 12 hour before the procedure. In the CT room, operation area was routinely sterilized and covered with sterile towel, local anesthesia with 2% lidocaine. After determined the location of the tumor in the CT scan, the seed implanted needles were inserted into the tumor accurately. When the needle tip reaches the deepest point of the tumor (about 1 cm away from the edge of the tumor), we take out the needle core. After aspiration without blood, the particles were pushed into the tumor by a thruster. In accordance with the principle of equidistance, the particles were implanted sequentially. The spacing of particles was about 0.5 to 1.0 cm. So the radiation source was arranged in a straight line. If there were more than 1 radioactive source, the radiation sources were relatively parallel, and the implanted needles were separated by 1cm. The distance between vessels, pancreatic duct and other important organs was at least 1cm, thus reducing puncture and radiation damage. The mean MPD in the group A was 120 Gy (100–140 Gy), and the median number of particles was 35 (15–60) while the mean MPD in the group C was 120 Gy (100–150 Gy), and the median number of particles was 41 (20–80). After surgery, the patient should stay in bed and fast for 6 hour. The blood amylase and urine amylase were examined the second days after operation. The intraoperative image was input into the TPS within 3 days after surgery to validate the dose again. Implantation was repeated in the “cold spot.” Surgeons worn lead clothing, scarf, gloves and glasses in order to avoid radiation damage. After the operation, a radioactive detector was used to detect if there was residual particles, so as to avoid radioactive contamination.

### 2.4. Arterial infusion chemotherapy

Operation area was routinely sterilized and covered with sterile towel, local anesthesia with 2% lidocaine. The right femoral artery puncture was performed with Seldinger technique. The 4~5 F RH or C3 catheter was selectively inserted into the celiac artery and superior mesenteric artery for the angiography. Splenic angiography was used at the same time to observe the blood supply of the pancreatic body or tail. Carcinoma of pancreatic head always selected gastroduodenal artery or superior mesenteric artery angiography, while carcinoma of pancreatic body or tail often selected the celiac artery or the splenic artery. Arterial infusion chemotherapy was performed according to the location of the tumor. Hepatic artery infusion chemotherapy was performed simultaneously in patients with liver metastasis. The potential infusion artery was carefully evaluated prior to the surgical procedure. The tumor with rich blood supply was treated with lipiodol embolization and infusion chemotherapy. The drugs of infusion chemotherapy were gemcitabine (1000 mg/m^2^) and cisplatin (50 mg/m^2^) (gemcitabine + cisplatin, GP), once every 21 to 28 days. We treated the patient 2~4 times depending on the patient tolerance. The infusion chemotherapy would be stopped when the patient experienced an intolerable side reaction, tumor progression or voluntary abandonment. Jaundice occurred in 14 of the 72 patients. These patients underwent percutaneous transhepatic cholangial drainage or Endoscopic Retrograde Cholangio-Pancreatography before chemotherapy to reduce jaundice and improve liver function.

### 2.5. Follow-up and evaluation criteria

The postoperative was reexamination once a month for the first 3 months after the operation, then reviewed once every 3 months. The examination involved upper abdominal enhancement CT scan, serum levels of carbohydrate antigen 19-9 (CA19-9), routine blood, blood biochemistry, pain score, KPS and so on. According to the standard of Response Evaluation Criteria in Solid tumor 1.1, the CT scans were compared with those within 2 weeks before treatment. The effectiveness of therapy was evaluated using the following criteria: complete response, partial response, stable disease, progressive disease, and survival rate.

### 2.6. Statistical analysis

The data was analyzed using SPSS 22.0 statistics software. *P* < .05 was considered to be statistically significant. Analysis of Variance was applied to estimate the baseline-characteristics of the patients. Pearson chi-square test was applied to estimate Local control rate (LCR: complete response + partial response + stable disease), CA19-9 decline percentage, pain evaluation and KPS. Kaplan–Meier curves and Log - rank test were applied to estimate survival time and draw survival curve. Deaths from all causes are listed as events.

## 3. Results

### 3.1. Local control and survival

The follow-up time was 1 to 36 months. Comparison of clinical efficacy between the 3 groups were observed in Table 2. The LCR of group A and group C was significantly higher than group B (*P* < .001), with no statistical difference between group A and group C (*P* > .05). Pearson chi-square test showed statistical difference of the 3 groups (χ^2^ = 12.969, *P* = .044).

**Table 2:** Comparison of clinical efficacy between the 3 groups.

M	Group A,n (%)					Group B,n (%)					Group C,n (%)					P (LCR)			
	CR	PR	SD	PD	LCR	CR	PR	SD	PD	LCR	CR	PR	SD	PD	LCR	Group A&B	Group B&C	Group A&C	Group A&B&C
1	0 (0)	10 (43.5)	11 (47.8)	2 (8.7)	21/23 (91.3)	0 (0)	10 (37.0)	12 (44.4)	5 (18.5)	22/27 (81.5)	0 (0)	13 (59.1)	8 (36.4)	1 (4.5)	21/22 (95.5)				
3	1 (4.5)	9 (43.5)	10 (40.9)	2 (9.1)	20/22 (90.9)	0 (0)	7 (31.8)	9 (40.9)	6 (27.3)	16/22 (72.7)	1 (4.5)	13 (59.1)	7 (31.8)	1 (4.5)	21/22 (95.5)				
6	1 (4.5)	8 (36.4)	8 (36.4)	5 (22.7)	17/22 (77.3)	0 (0)	2 (14.3)	4 (28.6)	8 (57.1)	6/14 (42.9)	2 (9.5)	15 (71.4)	3 (14.3)	1 (4.8)	20/21 (95.2)	<0.001	<0.001	0.442	<0.001
12	0 (0)	2 (28.6)	2 (28.6)	3 (42.9)	4/7 (57.1)	0 (0)	1 (16.7)	1 (16.7)	4 (66.7)	2/6 (33.3)	1 (7.1)	8 (57.1)	3 (21.4)	2 (14.3)	12/14 (85.7)				
24	0 (0)	0 (0)	1 (50%)	1 (50%)	1/2 (50%)	0 (0)	0 (0)	0 (0)	2 (100)	0/2 (0)	0 (0)	0 (0)	2 (66.7)	1 (33.3)	2/3 (66.7)				
36	0 (0)	0 (0)	0 (0)	2 ()	0/2 (0)	0 (0)	0 (0)	0 (0)	2 (100)	0/2 (0)	0 (0)	0 (0)	0 (0)	1 (100)	0/1 (0)				

CR = complete response, LCR = local control rate, PD = progressive disease, PR = partial response, SD = stable disease.

**Figure 1.**
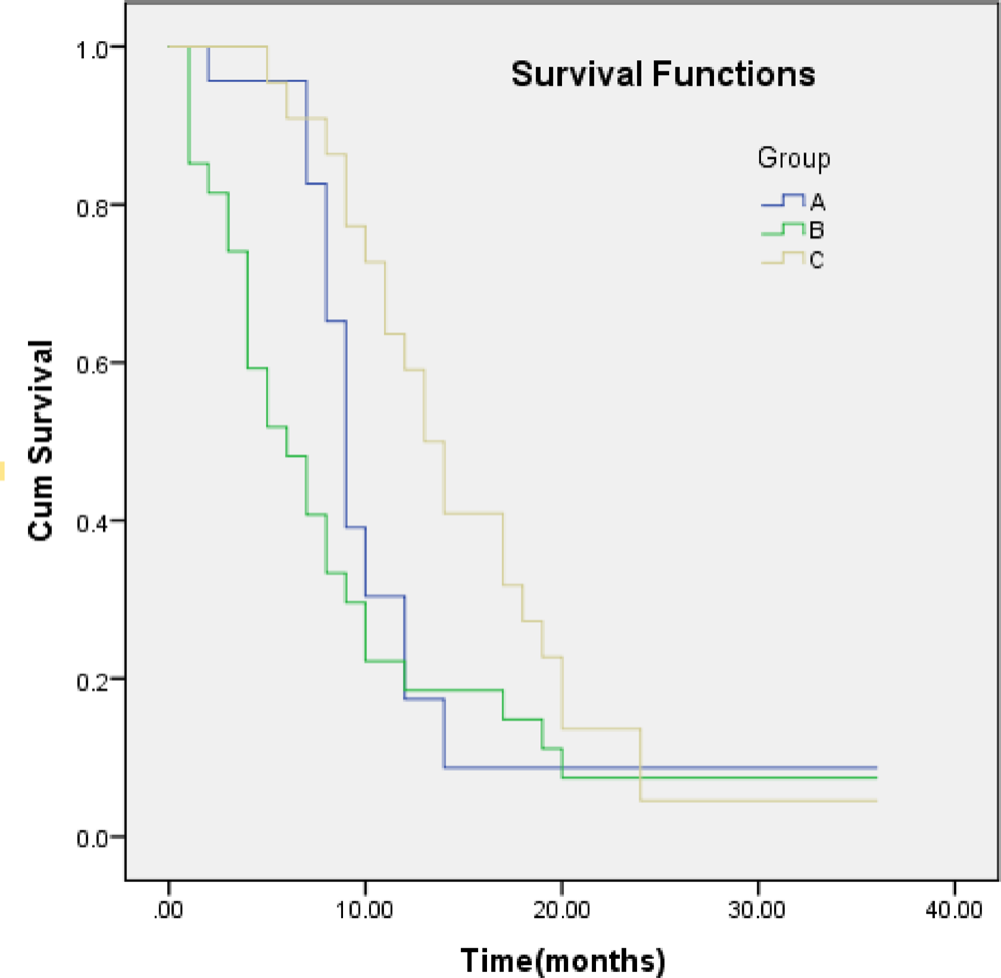
Survival functions of the 3 groups.

Kaplan–Meier curves was applied to estimate survival time and draw survival curve (Fig. 1). The median survival of group A, B and C was 9 months, 6 months and 13 months, respectively. The survival rate of the 3 groups were observed in Table 3. The survival time of group C was significantly higher than group B (χ^2^ = 5.403, *P* = .020), with no statistical difference between group A and group C, group B and C (*P* > .05). The Log rank test showed statistical difference in the survival curve of the 3 groups (χ^2^ = 6.501, *P* = .039).

**Table 3:** Survival rate of the 3 groups.

Survival time (m)	Group A		Group B		Group C	
	Cases	%	Cases	%	Cases	%
1	23	100%	27	100%	22	100%
3	22	95.7	22	81.5%	22	100%
6	22	95.7%	14	51.9%	21	95.5%
12	7	30.4%	6	22.2%	14	63.6%
24	2	8.7%	2	7.4%	3	13.6%
36	2	8.7%	2	7.4%	1	4.5%

### 3.2. CA19-9

CA19-9 is the marker with the most diagnostic value of pancreatic cancer. Within 1 week before the treatment, CA19-9 was determined for the 3 groups, with the normal reference value of 0 to 27 U/mL. The cases of CA19-9 higher than the upper limit value of the normal cases of Group A, group B and group C were 20 cases, 22 cases, 20 cases. After treatment, the CA19-9 decline percentage of Group A, group B and group C was 80.0% (16/20), 59.1% (13/22) and 90.0% (18/20). The difference of CA19-9 decline percentage between group B and C group was statistically significant (χ^2^ = 5.959, *P* = .015), the difference between group A and group B and the difference between group A and group C didn’t reach statistical significance (*P* > .05).

### 3.3. Pain evaluation and KPS

59 patients were in pain before treatment. The pain was graded by the VAS pain rating criteria. 0 point was no pain. 3 points and 3 points below were mild pain that could be tolerable. Total 4 to 6 points were moderate pain which influenced sleep but can be tolerable. 7 to 10 points were severe pain that were difficult to tolerate and affected appetite and sleep. The pain scale changed was shown in Table 4. Patients in group A and group C relieved form pain after treatment and the differences were statistically significant (*P* < .001), while that in group B was not statistically significant (*P* > .05). The pain of each group before treatment showed no statistical difference (*P* > .05). While, the pain after treatment showed statistical difference (the X^2^ between group A and group B was 9.027, *P* = .029, the X^2^ between group A and group C was 9.165, *P* = .0027, the χ^2^ between group B and group C was 24.656 [*P* < .001]).

**Table 4:** Changes of pain score and KPS.

Changes	^125^I brachytherapy		Arterial infusion		^125^I brachytherapy + arterial infusion	
	Before treatment (%)	After treatment (%)	Before treatment (%)	After treatment (%)	Before treatment (%)	After treatment (%)
Pain						
No pain	4/23 (17.4)	10/23 (43.5)	7/22 (25.9)	8/27 (29.6)	2/22 (9.1)	14/22 (63.6)
Mild pain	2/23 (8.7)	7/23 (30.4)	4/27 (14.8)	7/27 (25.9)	3/22 (13.6)	4/22 (18.2)
Moderate pain	9/23 (39.1)	4/23 (17.4)	7/27 (25.9)	6/27 (22.2)	5/22 (22.7)	2/22 (9.1)
Severe pain	8/23 (34.8)	2/23 (8.7)	9/27 (33.3)	6/27 (22.2)	12/2 (54.5)	2/22 (9.1)
KPS						
60	5/23 (21.7)	0/23 (0)	6/27 (22.2)	1/27 (3.7)	5/22 (22.7)	0/22 (0)
70	9/23 (39.1)	1/23 (4.3)	10/27 (37.0)	5/27 (18.5)	7/22 (31.8)	2/22 (9.1)
80	8/23 (34.8)	13/23 (56.5)	11/27 (40.7)	15/27 (55.6)	9/22 (40.9)	9/22 (40.9)
90	1/23 (4.3)	8/23 (34.8)	0/27 (0)	6/27 (22.2)	1/22 (4.5)	10/22 (45.5)
100	0/23 (0)	1/23 (4.3)	0/27 (0)	0/27 (0)	0/22 (0)	1/22 (4.5)
Mean	72.2	83.9	71.9	79.6	72.7	84.5

125 I = iodine-125, KPS = Karnofsky physical scores.

KPS after treatment were statistically higher than those before treatment in each group (*P* < .001). There was no statistically significant difference in KPS between each group after treatment (χ^2^ = 5.827, *P* = .054) (Table 4).

### 3.4. Complications

Follow-up results of the treatment: blood and urinary amylase of 7 patients increased, but within normal range. One seed of a patient moved to the liver. 7 patients had gastrointestinal symptoms. 2 patients had fever. Blood sugar of 3 patients increased but took a turn for the better after symptomatic treatment. 6 patients had mild decline of leukocyte count, which returned to normal after 1 month. There were no serious complications such as pancreatitis, fistula, abscess, bleeding, perforation, blood vessel and organ damage.

## 4. Discussion

The incidence of pancreatic cancer is still on the rise, with poor prognosis and is expected the fourth death rate of all the malignant tumors.^[1–3]^ The median survival time for untreated patients is 4 months, and they will suffer in varying degrees from pain, anorexia, weight loss, jaundice, and intestinal obstruction.^[4,5]^ The surgery of pancreaticoduodenal resection is the mainly treatment. Since its early symptoms are not obvious, most of the patients was in advanced and have distant metastasis that could not be removed surgically when diagnosed.^[4,5,15]^ The traditional conventional external beam radiation therapy can’t obtain satisfactory effects for pancreatic cancer. The main reason is that the pancreas is located in the deep belly, with liver, kidney, stomach and intestines around. These important viscus were radiation intolerance. Conventional radiation dose, in fact, which bases on the acceptable dose of normal tissue, is far less from tumor regression dose.

Compared with the conventional external radiotherapy, ^125^I radioactive particle as a brachytherapy has its unique advantage. The ^125^I radiation range is small and precise with high local treatment dose, high-dosed radiation increased local tumor control and OS.^[8–10]^ A computer 3-dimensional TPS and CT-guided imaging can accurately position particle placement position and help peripheral tumor doses reach the MPD of 100 to 150 Gy and reduce the risk of local recurrence. Dose distribution around the source decreased according to the distance of radioactive sources by the inverse square law. The dose outside the target volume fell down rapidly, leaving a slight injury to the surrounding tissues and organs. ^125^I has a long half-life of 59.6 days. So a highly effective dose of radiation as a single source can effectively kill tumor cells over long periods of time, lasting for weeks or months.^[9–11,16–20]^ Compared with traditional surgery, the seed implantation is simpler and can reduce the operative time, with smaller surgical trauma, less physical and mental pain, fewer complications and lower costs.^[21]^ In addition, the particles are easy to be preserved. The operator was under strict protection during the operation. The dose distribution was not affected by the movement of patients and their internal organs. The shell of the radioactive ^125^I particle is made of titanium alloy which is highly compatible with the human body and can be perpetual existence in the body without causing rejection.^[16–19,22]^ Arterial infusion chemotherapy is another treatment with minimally invasive of tumor. Chemicals are delivered directly into the target artery of the tumor. It can increase the drug concentration at the tumor and play a more effective antitumor effect. GP is the mainstay of chemotherapy for advanced pancreatic cancer, and the combination chemotherapy compared to gemcitabine alone significantly improves OS in advanced pancreatic cancer.^[11–14]^ However, there were few reports about ^125^I brachytherapy combined with chemotherapy for pancreatic cancer.

^125^I brachytherapy combined with arterial infusion chemotherapy of pancreatic cancer can better improve the patient quality of life and improve the LCR, median survival, survival rate and reduce CA19-9 level. Some of the benefits of ^125^I brachytherapy combined with arterial infusion chemotherapy also found in other cancers.^[23–25]^ In this study, the ^125^I brachytherapy combined with arterial infusion chemotherapy could achieve a median survival time of 13-month. LCR, median survival and survival rate were similar to previously reports.^[24–26]^ Implanted particles can kill a large number of cancer cells within the implanted area. Because of the reduced tumor burden, it may increase the sensitivity of these cells to chemotherapy drugs. To a certain extent, gemcitabine and cisplatin as radiation sensitizer can increase the radiation effect of ^125^I seeds, which have been found in advanced cholangiocarcinoma.^[25]^ Patients with pancreatic cancer frequently present with pain as the initial symptom, with nearly 80% of patients suffering from pain at the time of diagnosis.^[20,27]^ Pain relieved by ^125^I seed had also been previously reported.^[17,20]^ In the study, pain relief was much more effective in patients who received ^125^I brachytherapy. It might be related to block related celiac nerve plexus and the effect on secretion were alleviated and modulated.^[25]^ Higher CA19-9 serum levels were significantly associated with more malignant and the worse prognosis of pancreatic cancer.^[28]^ In the study, CA19-9 decline percentage was much more obvious in patients who received ^125^I brachytherapy combined with arterial infusion chemotherapy, which was consistent with the improvement of the LCR, median survival and survival rate.

Liver is the most frequent site for distant metastasis as well as recurrence of pancreatic cancer. In this study, stage IV patients with pancreatic cancer was 76.4%, in which the number of liver metastases cases was 51.4%. Meanwhile, arterial infusion chemotherapy of proper hepatic artery or transcatheter arterial chemoembolization for the patients with liver metastasis, can control the primary tumors and metastases at the same time.

Nowadays, only a few therapeutic options are available for pancreatic cancer, with poor prognosis, especially for the advanced stages. The OS in systemic chemotherapy or arterial infusion chemotherapy alone are all poor of advanced pancreatic cancer (2.7–14 vs 5–21 months).^[13,14,29,30]^ The suboptimal survival of the group B was roughly consistent in these studies with the same technique.^[13,14,29,30]^ As a pancreatic head tumor mass has blood supplies from both the superior and inferior pancreaticoduodenal arteries, local chemoagents infusion from the celiac or superior mesenteric artery fails to cover the whole lesion. The treatment effect may be even lower than that of aortic infusion.^[13]^ Molecular-targeted therapy and immunotherapy might provide more treatments in the future. Monotherapy inhibiting a single target is unlikely to produce a remarkable clinical benefit, and a combination of multiple targeted drugs might provide further opportunities for treatment.^[31–33]^

Despite multiple deficiencies and a small sample size, ^125^I brachytherapy maybe one of the effective, safe and feasible alternative treatment of advanced pancreatic cancer. ^125^I brachytherapy combined with arterial infusion chemotherapy was effective in the treatment of advanced pancreatic cancer.

## Acknowledgments

Thanks to all patients who took part in this clinical research.

## Author contributions

**Conceptualization:** Shujing Huang, Shu Yang.

**Data curation:** Shujing Huang, Yanqing Cao, Huimin Liu, Ting Wang.

**Formal analysis:** Shujing Huang, Yanqing Cao, Rui Wang, Shu Yang.

**Methodology:** Shujing Huang, Yanqing Cao, Rui Wang, Huimin Liu.

**Software:** Shujing Huang, Rui Wang.

**Supervision:** Shu YangValidation: Ting Wang.

**Writing – original draft:** Shujing Huang, Huimin Liu.**Writing – review & editing:** Shu Yang.
